# Obscurin Maintains Myofiber Identity in Extraocular Muscles

**DOI:** 10.1167/iovs.65.2.19

**Published:** 2024-02-09

**Authors:** Abraha Kahsay, Nils Dennhag, Jing-Xia Liu, Hanna Nord, Hugo Rönnbäck, Anna Elisabeth Thorell, Jonas von Hofsten, Fatima Pedrosa Domellöf

**Affiliations:** 1 Department of Integrative Medical Biology (IMB), Umeå University, Umeå, Sweden; 2 Department of Clinical Sciences, Ophthalmology, Umeå University, Umeå, Sweden

**Keywords:** obscurin, extraocular muscles, myofiber, myosin heavy chain 7, zebrafish

## Abstract

**Purpose:**

The cytoskeleton of the extraocular muscles (EOMs) is significantly different from that of other muscles. We aimed to investigate the role of obscurin, a fundamental cytoskeletal protein, in the EOMs.

**Methods:**

The distribution of obscurin in human and zebrafish EOMs was compared using immunohistochemistry. The two *obscurin* genes in zebrafish, *obscna* and *obscnb,* were knocked out using CRISPR/Cas9, and the EOMs were investigated using immunohistochemistry, qPCR, and in situ hybridization. The optokinetic reflex (OKR) in five-day-old larvae and adult *obscna^−/−^;obscnb^−^^/^^−^* and sibling control zebrafish was analyzed. Swimming distance was recorded at the same age.

**Results:**

The obscurin distribution pattern was similar in human and zebrafish EOMs. The proportion of slow and fast myofibers was reduced in *obscna^−^^/^^−^;obscnb^−^^/^^−^* zebrafish EOMs but not in trunk muscle, whereas the number of myofibers containing cardiac myosin *myh7* was significantly increased in EOMs of *obscurin* double mutants. Loss of obscurin resulted in less OKRs in zebrafish larvae but not in adult zebrafish.

**Conclusions:**

Obscurin expression is conserved in normal human and zebrafish EOMs. Loss of obscurin induces a myofiber type shift in the EOMs, with upregulation of cardiac myosin heavy chain, *myh7*, showing an adaptation strategy in EOMs. Our model will facilitate further studies in conditions related to obscurin.

The extraocular muscles (EOMs) differ significantly from other skeletal musculature. They consist of diverse myofibers that express a multitude of myosin heavy chain genes,^1^^–^^7^ which allow them to control very precise movements. Furthermore, we have shown that a subset of myofibers in both human and zebrafish EOMs lack desmin,^8^^,^^9^ which was previously considered ubiquitous in all muscles.

Obscurin is a gigantic sarcomeric Rho guanine nucleotide exchange factor protein of the titin family^10^^–^^14^ and is responsible for the assembly and maintenance of the stability of the muscle sarcomere.^15^^–^^17^ In humans, obscurin is abundantly expressed in skeletal and cardiac muscle and is localized at the Z-disc and M-band in the sarcomeres of developing muscle and only at the M-band in adult muscle.^18^ Obscurin is also abundantly localized in the vicinity of neuromuscular junctions (NMJs) and of myotendinous junctions (MTJs) of skeletal muscle where it is thought to interact with the submembrane cytoskeleton and regulate cellular architecture, stabilizing contacts between the myofiber and the extracellular matrix.^19^ Obscurin interacts with other muscle proteins such as titin, myomesin, and myosin, proteins that are essential for the proper organization of the sarcomeres and function of myofibers, in particular the assembly and maintenance of A and M bands.^18^^,^^20^^–^^23^ Additionally, obscurin is important to keep the alignment of the sarcoplasmic reticulum with the contractile apparatus.^24^^–^^26^

In humans, mutations in the *obscurin* gene (OBSCN) are associated with a range of muscle disorders, including hypertrophic and dilated cardiomyopathy, and limb girdle muscular dystrophy.^27^ These mutations result in altered obscurin expression and localization leading to impaired muscle development and function.^28^^,^^29^ In mice, lack of obscurin affects localization of dystrophin, as well as M-line organization.^30^^,^^31^

Obscurin is well conserved in different animal models, such as *Drosophila*, *Caenorhabditis elegans*, and zebrafish.^14^^,^^32^^,^^33^ Because of its high genetic homology with humans, zebrafish has become a novel and robust animal model to study muscle biology and disease.^34^^,^^35^ However, unlike mammalian obscurin, which is encoded by a single gene, zebrafish obscurin is encoded by two genes, *obscna* and *obscnb* located on zebrafish chromosomes 8 and 24, respectively, because of a genome duplication event early in evolution.^33^ During development, zebrafish *obscna* is expressed in somites, central nervous system and the heart and has been proposed to play an important role in striated myofibril assembly.^33^ However, the expression and function of *obscnb* has not yet been examined.

Even though the role of obscurin is well documented in human trunk, cardiac, and diaphragm muscles, the role of obscurin in EOMs is essentially unknown. Here, we demonstrate the expression pattern of obscurin in human and zebrafish EOMs, including the NMJs and MTJs. Furthermore, we demonstrate that lack of obscurin resulted in functional alterations and myofiber type shifts specific to the EOMs.

## Material and Methods

### Human Material

Human EOM samples were collected 14 to 48 hours postmortem from eight male subjects (mean age 70 years, range 42–84 years). All experiments were approved by the Regional Ethical Review Board in Umeå and conducted in accordance with the principles of the Declaration of Helsinki. The muscles were obtained from deceased individuals who, when alive, had consented to donate their eyes and other tissues postmortem for transplantation and research purposes, according to Swedish law. None of the control subjects had a history of neuromuscular disease. Upon collection, EOM samples were rapidly transported, carefully protected, on ice, oriented and mounted on cardboard, rapidly frozen in propane chilled with liquid nitrogen, and stored at −80°C until sectioned. Serial cross- and longitudinal sections (7 µm thick) were cut in a cryostat (Reichert Jung; Leica, Nussloch, Germany) and kept at −20°C until processed for immunohistochemistry.

Additional muscle samples were stretched on a cork-plate and fixed for one hour in freshly prepared 2% paraformaldehyde (PFA) in 0.1 M PBS containing 0.01% glutaraldehyde (GA), rinsed in 0.1 M PBS, and then treated in 10% sucrose at 4°C overnight. Thereafter, samples were retreated in 20% sucrose and stored −80°C until used. The muscle samples were thawed and cut into small pieces and infiltrated in 2.3 M sucrose at 4°C overnight. Specimens were longitudinally oriented and frozen in liquid nitrogen, and 1 µm thick sections were cut with a Reichert Ultracut microtome, equipped with FCS cryo attachment (Leica).

### Human Immunohistochemistry

The rabbit polyclonal antibody obscurin IQ was used to detect the binding motif for calmodulin-like obscurin protein^36^ (Gift from Prof. M. Gautel, King's College London, UK). A mouse monoclonal antibody against the gamma subunit acetylcholine receptor (AChRγ; GTX74890; Gentex, Landskorna, Sweden) was used to identify NMJs, and a sheep polyclonal antibody against laminin (PC128; Binding Site Group, Birmingham, UK) was used to identify MTJs and myofiber contours. Tissue sections were processed for double immunolabeling to visualize obscurin in myofibers, and their NMJs and MTJs. In brief, sections were air-dried for 20 minutes, rehydrated in 0.01M PBS, blocked with 5% donkey serum, and incubated with mixed primary antibodies against obscurin, myomesin, laminin, and AChRγ (Supplementary Table S1) overnight at 4°C. The procedure of rehydration with 0.01M PBS and blockage with 5% donkey serum was repeated on the following day and secondary antibodies raised from appropriate hosts and conjugated with different fluorochromes (Supplementary Table S2) or directly conjugated high-affinity probes (Supplementary Table S3) were used for incubation at 37°C for 30 minutes. The samples were treated in 0.01 M PBS and finally mounted with Vectashield mounting medium (Vector Laboratories, Inc., Burlingame, CA, USA). The 1 µm thick EOM sections were photographed using Nikon confocal microscopy (Nikon, Tokyo, Japan) whereas 7 µm-thick sections were examined and photographed under a Leica microscope (Leica DM 6000 B; Leica Microsystems, Wetzlar, Germany), equipped with a digital camera (Leica DFC360 FX; Leica Microsystems). The images were processed using Adobe Photoshop software (Adobe System, Inc., Mountain View, CA, USA).

### Zebrafish Maintenance

Zebrafish (Danio rerio) were maintained at the Umeå University Zebrafish Facility. All animal experiments were approved by the Regional Ethics Committee at the Court of Appeal of Northern Norrland, Dnr A6 2020. Zebrafish embryos were raised at 28.5°C in embryo (E3) medium and staged in days postfertilization (dpf).

### Generation of *Obscna* and *Obscnb* Knockout Zebrafish Lines

Zebrafish mutants were generated using CRISPR/Cas9 gene editing technology. Single guide RNAs (sgRNAs) targeting the coding region of both *obscna* (XM_017357438.2) and *obscnb* (XM_021470383.1) zebrafish genes, 5ʹ-TCAAGTAATGTCCGGACG-3ʹ and 5ʹ-ATGGATCAGAATCTATTT-3ʹ were designed using online gRNA design tool by Sigma Aldrich Merck CRISPR (https://www.milliporesigmabioinfo.com/bioinfo_tools/faces/secured/crispr/crispr.xhtml). Single-cell zebrafish embryos were microinjected with a mixture of sgRNA and Cas9 protein. Injected zebrafish embryos were raised to adult age and out crossed with wild-type fish, and the adult F1 generation were fin clipped and genotyped for germline transmission. To extract genomic DNA, fin clips or whole embryos were lysed with 25 µL of NaOH (50 mM), incubated at room temperature for one hour and denatured at 98°C for 10 minutes in thermocycler. Genomic DNA 1 µL was used to perform PCR amplification using GoTaq G2 DNA Polymerase (Thermo Fisher Scientific, Waltham, MA, USA). Targeted region of *obscna* and *obscnb* were amplified using the forward (F) and reverse (R) primers; *obscna*_F: 5ʹ-TGATGATGTTGCCCAGTGTT-3ʹ, *obscna*_R: 5ʹ-TTCTTTCCATGGGTCTCTGC-3ʹ, and *obscnb*_F:5ʹ-CAACAAAGGTAACAAACAAACCA-3ʹ,*obscnb*_R:5ʹ AGGTACAGGGTTTCCCACAA-3ʹ followed by restriction digestion. The wild-type *obscna* PCR product contains a BbvCI (New England BioLabs, Ipswich, MA, USA) restriction site (in the wild-type sequence). The CRISPR/Cas9 mutagenesis resulted in loss of the BbVCI site, which was subsequently used to identify wild-type versus *obscna* mutant alleles. For identification of *obscnb* alleles, a SacI (Thermo Scientific, Vilnius, Lithuania) restriction site was utilized in a similar strategy.

### Optokinetic Reflex

The optokinetic reflex (OKR), was recorded using the Viewpoint zebrafish VisioBox system. Zebrafish larvae were mounted in 3% methyl cellulose, dorsal side up, inside a 3 cm diameter petri dish. The dish was then placed inside a 7.5 cm-diameter striped drum rotating at 0.04 cycles per degree (cpd), equivalent of 2 cm per cycle, the optimum speed and cpd for 5 dpf larvae.^37^ Each larva was subjected to OKR testing for 120 seconds. Each larva was individually placed in PBS to wash away methyl cellulose and subsequently genotyped for *obscna* and *obscnb,* as described above. The number of OKRs, each defined as a slow phase followed by a fast phase, were quantified using the Viewpoint ZebraLab software. OKRs in adult zebrafish was evaluated using an adapted method,^38^ where a custom made OKR device was constructed with a rotating drum and interchangeable spatial frequency gratings. The speed of the rotating drum was controlled by an adjustable power supply run at a constant (5 Volts) to achieve 0.04 cpd. The 16-month-old zebrafish were wrapped in wet Kleenex towels and secured between two sponges tightened together with needles and placed in a container filled with system water in the center of the rotating drum, rendering them unable to move their body yet capable of moving their eyes and allowing gill movement. A high-speed camera was used to capture video recordings of adult zebrafish OKRs (Supplementary Video S1), and OKRs were manually counted at 50% recording speed over a 120 seconds period.

### Swimming and Resistance Analysis

The 5 dpf zebrafish larvae were placed in a 48 well-plate inside the Viewpoint ZebraBox system (Viewpoint Behavior Technology, Civrieux, France), and adult zebrafish were placed in a zebrafish breeding tanks on top of the Viewpoint ZebraTower system (Viewpoint Behavior Technology, Civrieux, France) to determine spontaneous movement patterns. Both experiments were analyzed in the Viewpoint ZebraLab software. The movement thresholds were set to inactivity = 0 and large movements = 1. Inactivity, small movement and large movement counts, swimming distance, and swimming duration were recorded. For resistance swimming, 4 dpf zebrafish larvae were incubated at different concentrations (0.6%, 0.8%, and 1%) methylcellulose in E3 medium and incubated at 28.5°C overnight. The following day, larvae were analyzed using birefringence.

### Cryosectioning

The head and trunk part of adult zebrafish, 10 months of age were fixed in 4% PFA for 2 h at room temperature, washed in PBS, dehydrated in 10% sucrose overnight and transferred to 30% sucrose for overnight incubation. The tissue was then embedded in OCT, frozen and stored at −80°C until sectioned at a thickness of 14µm for EOMs and 10µm for trunk using a cryostat (Reichert Jung; Leica, Heidelberg, Germany). The sections were stored at −20°C until use.

### Zebrafish Immunohistochemistry

Slides with cross-sections of EOMs and trunk were washed in PBS and then pre-incubated in blocking buffer (1% DMSO and 5% sheep serum in PBS Triton) for at least 1h at room temperature. Pre-blocking solution was removed and replaced with primary antibodies (Supplementary Table S1) for incubation at 4°C overnight. Following PBS washing slides were incubated in secondary antibodies and directly conjugated high affinity probes (Supplementary Tables S2 and S3) at 4°C overnight. Unbound secondary antibody was washed out with PBS and slides were mounted in glycerol. Images were taken using a Nikon confocal microscopy (Nikon, Tokyo, Japan).

### Whole Mount In Situ Hybridization

RNA probes for in situ hybridization were synthesized as previously described.^39^ Zebrafish embryos, larvae, and dissected adult EOMs were fixed in 4% PFA, overnight at 4°C, and dehydrated in a series of 30%, 50%, 70%, and 100% methanol and stored in 100% methanol at −20°C until use. Whole mount in situ hybridization was performed as previously described.^40^ The RNA probes were based on the sequences of *myh7bb* (XM_009296759), *myh7l* (NM_001077464) and *myh7* (NM_001112733). Primers used were forward 5ʹ-GCGGCCACATTTCTGCGTAA-3ʹ and reverse 5ʹ-TGCTTCGCCAAGAGCAGCTA-3ʹ for *myh7bb*, forward 5ʹ-CAGGCTTGTGAAGGGCAAGC-3ʹ and reverse 5ʹ-TCCAGTTGGCCCAGAAGACC-3ʹ for *myh7l* and forward 5ʹ-TGAAGAGTCGCAAGCCGAGT-3ʹ and reverse 5ʹ-TCCTCGTGCTCCAGTGATGC-3ʹ for *myh7*.

### RNA Extraction and Quantitative PCR

Zebrafish larvae from wild type and *obscurin* double mutants were collected at 5dpf for RNA extraction. Only the head part, excluding the heart, of 10 zebrafish larvae were used to harvest RNA from each genotype using a method previously described.^41^ cDNA was synthesized using SuperScript IV (Invitrogen). Primers used were forward 5ʹ-GCCGTCGCTACAGTTTTTGG-3ʹ and reverse: 5ʹ-TTTGCCAGCACCGGATTCTC-3ʹ for *myh7bb* and forward 5ʹ-TGTTAAAGCCACCGTCGTGA-3ʹ and reverse 5ʹ-TGCTGGCTCATGGAGAAAGG-3ʹ for *myh7l* and forward 5ʹ-GACAAGGCAATCATGGGGGA-3ʹ and reverse 5ʹ-GAGGGTTCTGGGGGTGAATG-3ʹ for *myh7*. The β-actin was used as a reference gene using primers forward 5ʹ-GCCTTCCTTCCTGGGTATGG-3ʹ and reverse 5ʹ-CCAAGATGGAGCCACCGAT-3ʹ. The qPCR was performed with Applied Biosystems ViiA-7 Real Time PCR System (Applied Biosystems, Carlsbad, CA, USA) using FastStart universal SYBR green master mix (Roche Diagnostics GmbH, Mannheim, Germany).

### Data Analysis

All statistical analyses were performed using GraphPad Prism 9.3.1. Quantification of immunofluorescent myofibers of the EOMs was done using Fiji ImageJ (https://imagej.net/software/fiji/)^42^ and Adobe photoshop (Adobe System, Inc., Mountain View, CA, USA). Statistical analysis was conducted using a *t*-test with Welch's correction, one-way ANOVA followed by post hoc *t*-test, *P* ≤ 0.05 was considered significant (*P ≤ 0.05; **P ≤ 0.01; ***P ≤ 0.001; ****P ≤ 0.0001). Human data are presented as mean± standard deviation (SD) and zebrafish data are presented as mean ± standard error of mean (SEM). Significant differences between groups was set to alpha value <0.05.

## Results

### Obscurin Distribution Pattern Is Similar in Human and Zebrafish EOMs

**Figure 1.**
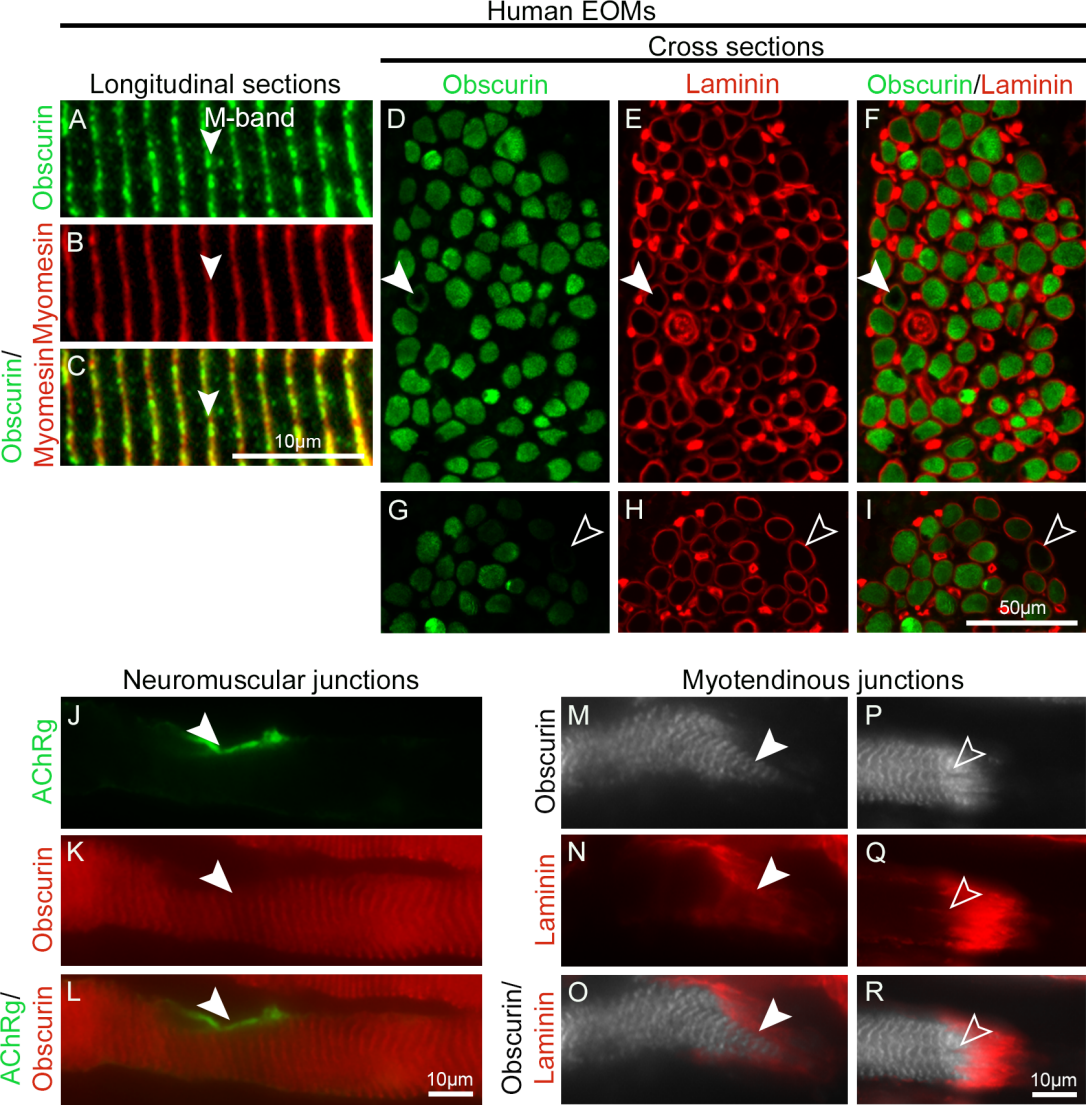
Localization and distribution of obscurin in human EOMs. (**A–C**) Longitudinal sections of human EOMs immunolabeled with antibodies against obscurin (*green* in **A**) and myomesin (*red* in **B**; merged in **C**), *arrowheads* indicate the M band. (**D–I**) Adjacent cross-sections of human EOM immunostained with antibodies against obscurin (*green* in **D** and **G**) and laminin (*red* in **E** and **H**; merged images in **F** and **I**). Note that obscurin was present in the vast majority of the human EOM myofibers. In a small percentage of myofibers, obscurin was present only subsarcolemally (*arrowheads* in **D–F**) or absent (*open arrowheads* in **G–I**). (**J–R**) Longitudinal sections of human EOM are shown. Obscurin (*red* in **K** and **L**) was maintained at the NMJs labeled by ACHRγ (*arrowheads*, *green* in **J** and **L**) rather than to be enriched, as known from the limb and trunk muscles.^19^ (**M–R**) Obscurin was also maintained (*arrowheads*, *gray* in **M** and **O**) in the majority of MTJs, but it was increased (*open arrowheads*, *gray* in **P** and **R**) at some MTJs. MTJs of human EOMs were identified by their localization and typical labeling by laminin (*arrowheads*, *red* in **N** and **O**, **Q** and **R**).

We first analyzed whether the spatial expression of obscurin is conserved in human and zebrafish EOMs, including in the vicinity of the neuromuscular junctions and at the myotendinous junctions of the myofibers. Using immunohistochemistry, we found obscurin to be widely distributed in human EOM myofibers (Figs. 1A–I), at NMJs (Figs. 1J–L), and in MTJs (Figs. 1M–R). In longitudinally sectioned human EOM myofibers, the obscurin immunolabeling was distinctly positioned in the M-band shown by co-labeling with myomesin (Figs. 1A–C) and in line with previous findings in human limb muscle.^19^^,^^36^ High levels of obscurin immunoreactivity were observed in the vast majority of human EOM myofibers (98.3% ± 0.3%) whereas a small portion of myofibers were labeled only subsarcolemmally (0.9% ± 0.5%, Figs. 1D–F) or had extremely low obscurin immunolabeling intensity (0.9% ± 0.3%, Figs. 1G–I, open arrowheads). The most abundant immunolabeling pattern (79% ± 17%) showed none or just negligible alteration of obscurin organization in the close vicinity of motor endplates (Figs. 1J–L). Obscurin immunolabeling was present in all MTJs (Figs. 1M–R). More than half of the MTJs examined (Figs. 1M–O, arrowheads) in human EOM myofibers had maintained staining intensity. Less than 40% of the myofibers showed increased labeling intensity with stronger striations at MTJs compared to the rest of the human EOM myofibers (Figs. 1P–R, open arrowheads) as described in other muscles.^19^

**Figure 2.**
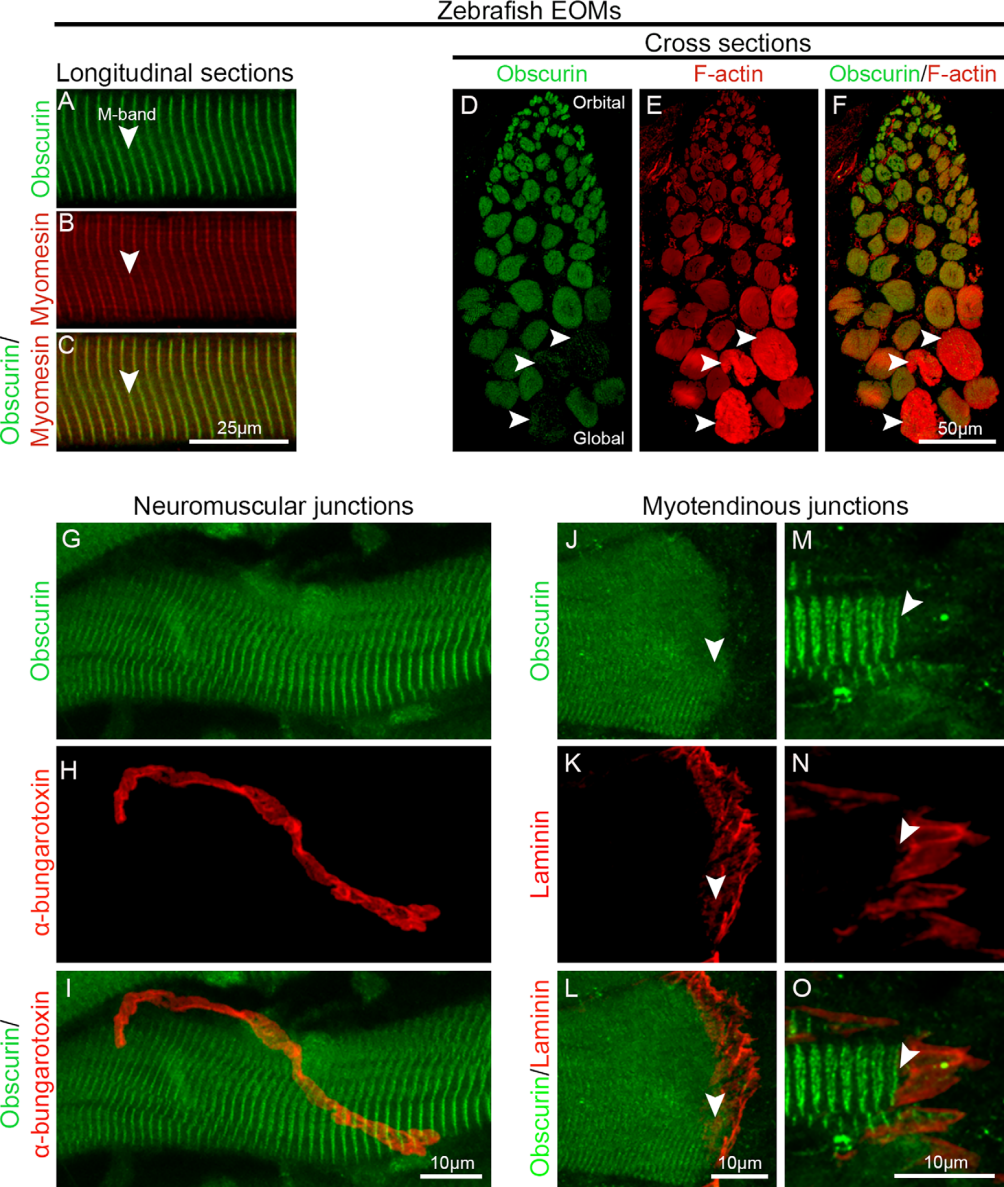
Obscurin distribution in zebrafish EOMs. (**A–C**) Longitudinal sections of 10-month-old wild-type zebrafish EOMs immunolabeled with antibodies against obscurin (*green* in **A**), and myomesin (*red* in **B**; merged image in **C**). Obscurin is localized in the M-band identified by staining with antibodies against myomesin (*red* in **B**). (**D–F**) Immunolabeling against obscurin (*green* in **D**) and F-actin (*red* in **E**; merged in **F**) showed the presence of obscurin in the vast majority of the myofibers in zebrafish EOMs. Lack of obscurin was also noted in a small subgroup of myofibers (*arrowheads*). Longitudinal sections of zebrafish EOMs immunolabeled with obscurin (*green* in **G**) at NMJs identified by α-bungarotoxin (*red* in **H**; merged in **I**) showed that obscurin was not enriched at NMJs as in other muscles. Immunolabeling of obscurin (*arrowheads*, *green* in **J**, **M**) at MTJs of zebrafish EOMs labeled by laminin (*red* in **K**, **N**; merged in **L** and **O**).

Obscurin was also localized at the M-band of the myofibers in the zebrafish EOMs (Figs. 2A–C) as in humans. Obscurin was present in the vast majority (93.55% ± 3.9%) of myofibers in zebrafish EOMs, even though a subset of myofibers in the global layer (6.4% ± 4.4%) exhibited extremely low obscurin intensity (Figs. 2D–F, arrowheads). The subcellular distribution of obscurin was uniform across the zebrafish EOM myofibers and their NMJs (Figs. 2G–I). Similar to the human EOMs, obscurin was present in all MTJs (Figs. 2J–O, arrowheads). Thus zebrafish and human EOMs obscurin immunolabeling patterns are similar. In summary, obscurin immunolabeling was much more uniform than that of other intermediate filament proteins in the EOMs^8^^,^^9^^,^^43^^,^^44^ in both human and zebrafish EOMs. Notably, obscurin was not enriched at NMJs or all MTJs, as expected from what is known from other muscles.

### Lack of Obscurin Alters Desmin Distribution in Zebrafish EOMs

**Figure 3.**
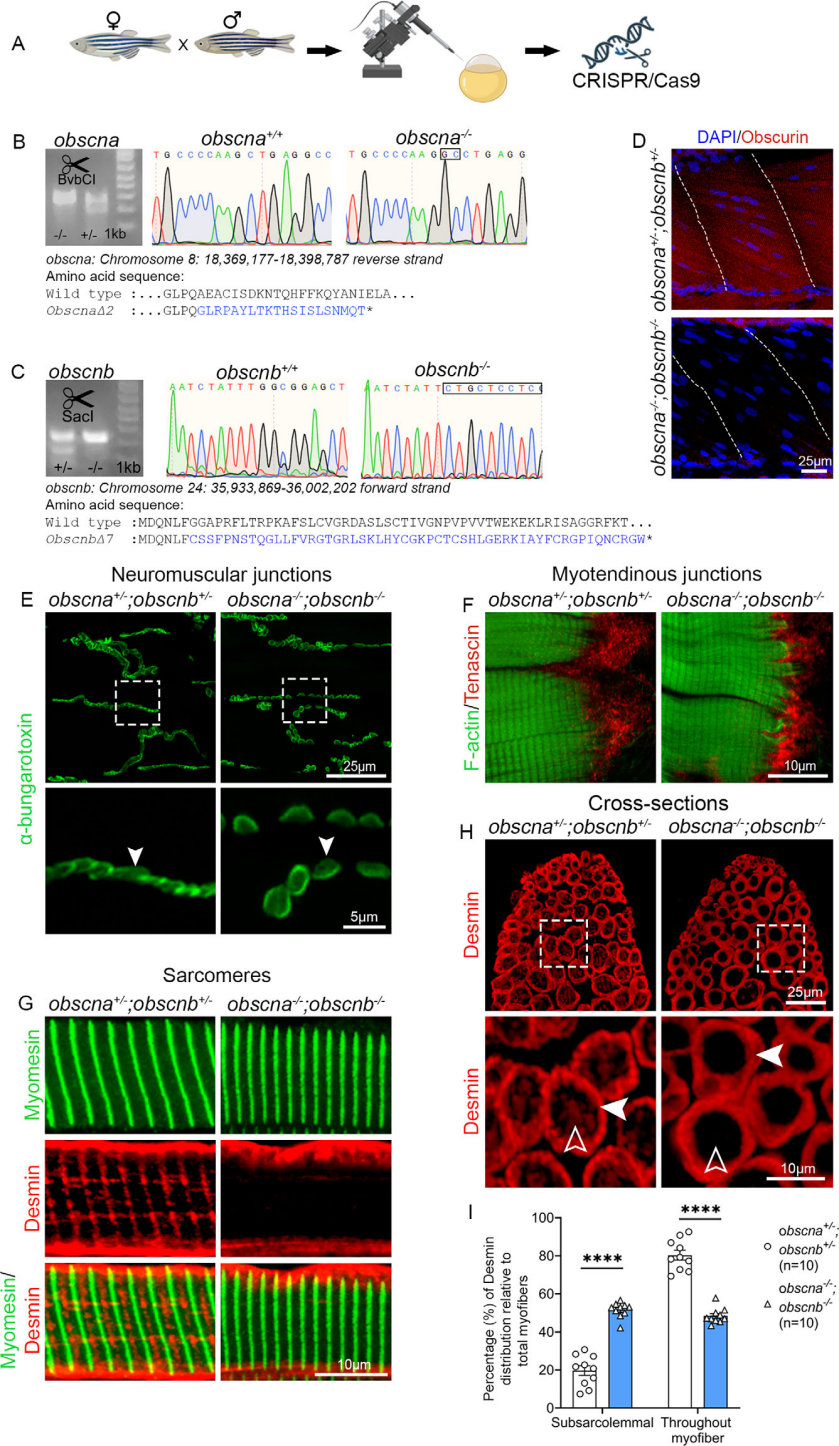
*Obscna* and *obscnb* knockout zebrafish (**A**) Schematic representation of generation of knockouts using CRISPR/Cas9 gene editing technology in zebrafish. (**B, C**) Amino acid sequence illustrating Obscna and Obscnb protein. *Blue letters* indicate the frameshift mutation resulting in premature stop codon indicated by *asterisk*. The resulting genotyped PCR products after cutting with restriction enzymes (*obscna*; BbVCI; New England BioLabs, Ipswich, MA, USA) and *obscnb;* SacI (Thermo Scientific, Vilnius, Lithuania) are shown using gel electrophoresis. DNA sequences of *obscna* and *obscnb* knockouts are displayed with chromatography, and mutation sites are shown in the box. (**D**) Obscurin immunolabeling of *obscna^+/^^−^;obscnb^+/^^−^* and *obscna^−^^/^^−^;obscnb^−^^/^^−^* zebrafish larvae at 5dpf. Longitudinal sections of 10-month-old zebrafish EOMs immunolabeled with (**E**) α-bungarotoxin labeling multiple nerve endings (*arrowheads*) in *obscna^+/^^−^;obscnb^+/^^−^* and *obscna^−^^/^^−^;obscnb^−^^/^^−^* adult zebrafish. (**F**) F-actin (*green*) and Tenascin (*red*) immunolabeling in whole EOMs of 10-month-old zebrafish showing MTJs of myofibers of *obscna^+/^^−^;obscnb^+/^^−^* and *obscna^−^^/^^−^;obscnb^−^^/^^−^* adult zebrafish. (**G**) myomesin (*green*) labeling M-band, desmin (*red*) labeling Z-discs (desmin-positive myofiber on the left column, and desmin-negative myofiber on the right column). (**H**) Cross-sections of 10-month-old zebrafish EOMs: subsarcolemmal localization of desmin in *obscna^−^^/^^−^;obscnb^−^^/^^−^* and distributed throughout the myofiber in *obscna^+/^^−^;obscnb^+/^^−^* (*arrowheads* indicate sarcolemma, *open arrowheads* indicate cytoplasm). (**I**) Percentage of myofibers showing desmin distribution either subsarcolemmaly or throughout the myofiber relative to the total number of myofibers. ****P < 0.0001.

To study the functional role of obscurin in zebrafish, both *obscna* and *obscnb* were knocked out using CRISPR/Cas9 (Fig. 3A). Genetic deletions leading to frameshifts and premature stop codons in both *obscna* and *obscnb*, were confirmed by sequencing and immunohistochemistry at 5 dpf showed lack of obscurin protein (Figs. 3B–D). Single heterozygous and homozygous mutants showed no major defects and developed to adult age (data not shown). Similarly, *obscna^−^^/^^−^;obscnb^−^^/^^−^* mutants survived to adulthood at mendelian ratios and no major morphological mutant phenotype could be identified (data not shown). Using α-bungarotoxin, we noted a subtle difference in the NMJs of EOMs of *obscurin* double mutants: a number of multiple separated junctional folds in the *obscurin* double mutant NMJs whereas such appearance of the NMJs was rarely noted in the sibling controls (Fig. 3E, arrowheads). We found no major difference in the MTJs of the zebrafish EOMs between *obscurin* double knockouts and their sibling controls (Fig. 3F). Given the proposed function of obscurin in assembly and maintenance of the stability of muscle sarcomere structure by interaction with proteins like titin and myomesin,^17^^,^^20^^–^^22^ we examined whether the sarcomeres and costameres were affected in the absence of obscurin. Longitudinal sections of zebrafish EOMs from *obscna^−^^/^^−^;obscnb^−^^/^^−^* and *obscna^+/^^−^;obscnb^+/^^−^* were immunolabeled with myomesin and desmin antibodies to identify the M band, the Z disc, and the subsarcolemmal part of the costameres of the myofibers, respectively. The M band was found well organized in *obscurin* double mutants and sibling controls (Fig. 3G, green). Interestingly, a high number of myofibers did not show positive desmin labeling in sarcomeric positions in the *obscna^−^^/^^−^;obscnb^−^^/^^−^* mutant zebrafish EOMs (Fig. 3G, red). On cross-sections, desmin was only present subsarcolemmally in 51.6% ± 1.21% of myofibers in *obscna^−^^/^^−^;obscnb^−^^/^^−^* compared to 19.7% ± 2.42% in the sibling controls (Figs. 3H-I, arrowheads, p < 0.0001). In addition, we found that the subsarcolemmal compartment containing desmin appeared thicker in the *obscna^−^^/^^−^;obscnb^−^^/^^−^* double mutants compared to sibling controls (Fig. 3H, arrowhead). In the sibling controls desmin was found in various patterns throughout the myofiber and subsarcolemmally (Figs. 3H, 3I) as previously reported.^9^ As innervation and myofiber types are interdependent,^45^ the altered NMJ patterning together with the phenotypic variation observed in *obscna^−^^/^^−^;obscnb^−^^/^^−^* mutant EOMs suggested a possible myofiber identity shift.

### Knockout of *Obscurin* in the EOMs Shifts Myofiber Identity in Zebrafish

**Figure 4.**
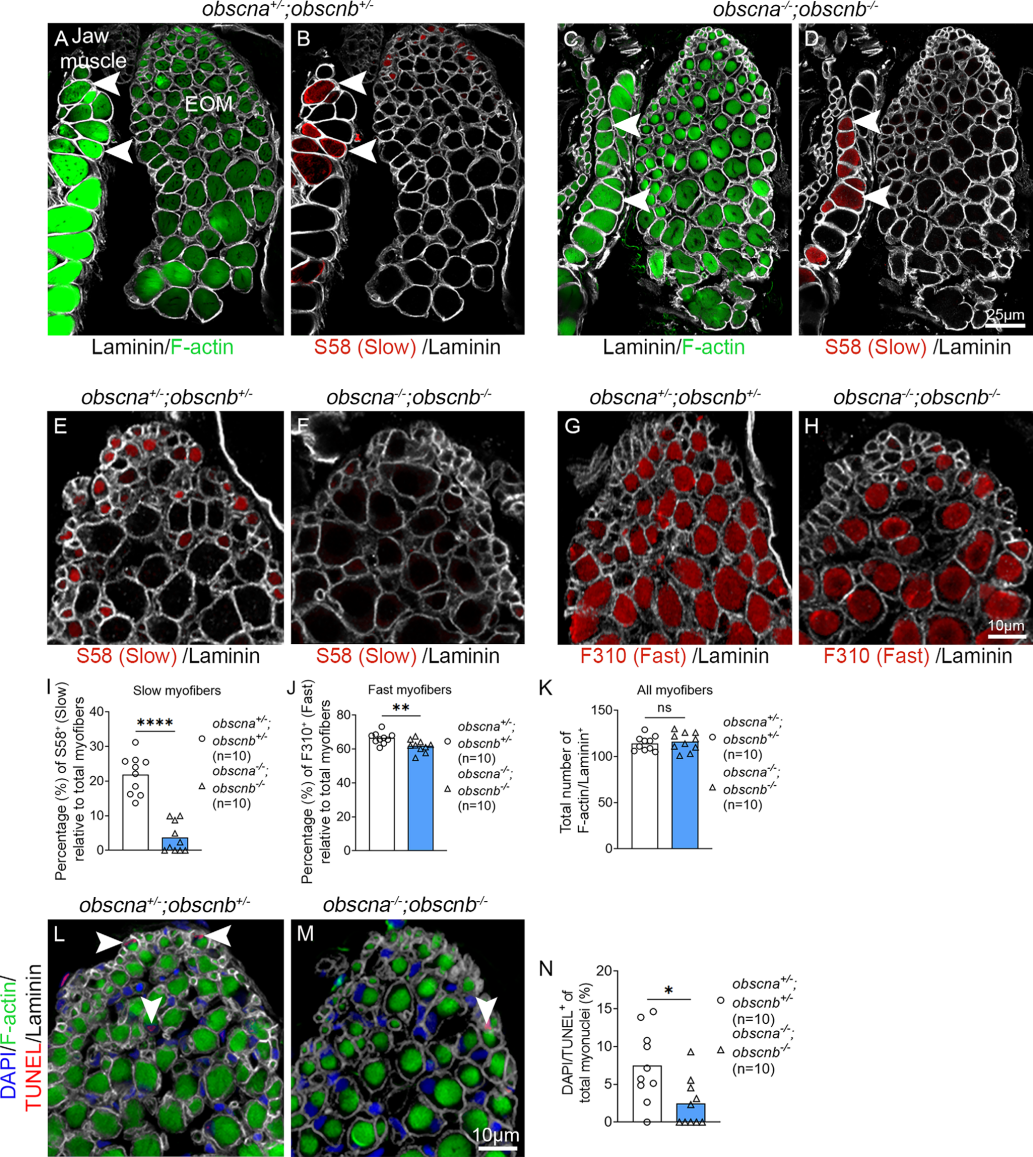
Quantification of slow and fast myofibers in the EOMs of adult obscurin mutants and sibling controls. Cross-sections of 10-month-old adult zebrafish EOMs immunolabeled with phalloidin to identify all myofibers (labeling of F-actin by phalloidin, *green* in **A** and **C**), S58 to identify all myofibers containing slow MyHC (*red* in **B**, **D–F**), F310 to identify all myofibers containing fast MyHC (*red* in **G** and **H**). The contours of the myofibers were labeled by the antibody against laminin (*gray* in **A–H**). *Arrowheads* indicate examples of positively-labeled myofibers with phalloidin, which labels F-actin (in **A** and **C**) and slow MyHC S58 (in **B** and **D**) in closely located masticatory myofibers (*left*) in the same section. Quantification of slow myofibers (S58 positive in **E** and **F** and quantified in **I**), fast myofibers (F310 positive in **G** and **H** and quantified in **J**), and the total number of myofibers (**K**) in *obscna^−^^/^^−^;obscnb^−^^/^^−^* and *obscna^+/^^−^;obscnb^+/^^−^*. (**L, M**) Cross-sections of *obscna^+/^^−^;obscnb^+/^^−^* and *obscna^−^^/^^−^;obscnb^−^^/^^−^* zebrafish EOMs immunolabeled with phalloidin, which labels F-actin, laminin, DAPI and TUNEL (*arrowheads*, *red* in **L** and **M**) to identify apoptotic myofibers. (**N**) The proportion of TUNEL-positive myofibers. Data in graphs are presented as mean ± SEM. *P < 0.05; **P < 0.01; ****P < 0.0001.

**Figure 5.**
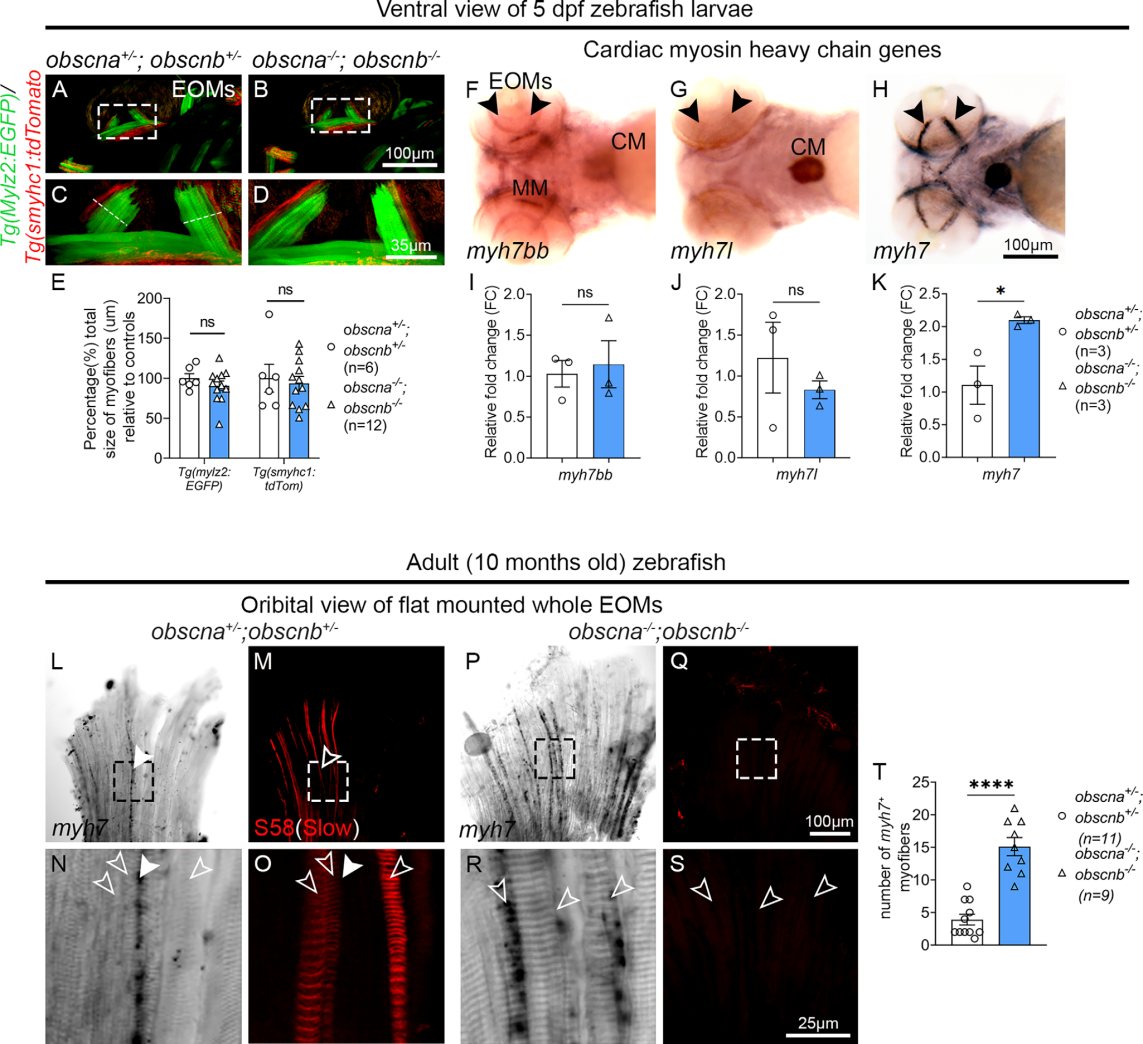
Expression of *myh7* myofibers of zebrafish EOMs. (**A–D**) Ventral view of the zebrafish EOMs (*dashed squares*) in double transgenic lines, *Tg(mylz2:*GFP, *green*, identifies all fast myofibers) and *Tg(smyhc:*tdTomato, *red*, identifies all slow myofibers) of *obscna^+/^^−^;obscnb^+/^^−^* and *obscna^−^^/^^−^;obscnb^−^^/^^−^* zebrafish larvae at 5 dpf. **C** and **D** show the EOMs in the marked areas in **A** and **B** at higher magnification. (**E**) Measurement of fast (identified by *Tg(mylz2:*GFP) and slow *Tg(smyhc:*tdTomato) myofibers total size (µm) in the EOMs relative to controls measured in midportion of the EOMs, indicated by *white line* in **C**, presented in percentage. (**F–H**) Ventral view of wild-type zebrafish larvae at 5dpf showing expression of cardiac myosin heavy chain genes, (**F**) myosin heavy chain 7bb (*myh7bb*), (**G**) myosin heavy chain 7-like(*myh7l*), and (**H**) myosin heavy chain 7 (*myh7*) probes in the EOMs and the cardiac muscle, *arrowheads* indicate EOMs, MM = masticatory muscle and CM = cardiac muscle. The qPCR showed the mRNA level of (**I**) *myh7bb,* (**J**) *myh7l*, and (K) *myh7* in *obscna^−^^/^^−^;obscnb^−^^/^^−^* and *obscna^+/^^−^;obscnb^+/^^−^* zebrafish larvae. Expression of *myh7* was significantly increased in EOMs. (**L–O**) EOMs from *obscna^+/^^−^;obscnb^+/^^−^* treated with *myh7* antisense probe showing small subgroup of *myh7*-positive myofibers (**L**, **N**, *arrowheads*), never overlapping with S58 labeled slow myofibers (**M**, **O**, *open arrowheads*) and (**P–S**) EOMs from *obscna^−^^/^^−^;obscnb^−^^/^^−^* where *open arrowheads* indicate *myh7*-positive myofibers. The areas indicated by the *squares* in **L**, **M**, **P**, and **Q** are shown below in higher magnification. (**T**) Quantification of the number of *myh7* positive myofibers in *obscna^−^^/^^−^;obscnb^−^^/^^−^* (n = 9) and *obscna^+/^^−^;obscnb^+/^^−^* (n = 11). *P < 0.05; ****P < 0.0001.

To identify a potential myofiber type shift in *obscna^−^^/^^−^;obscnb^−^^/^^−^* mutant zebrafish, the EOM cytoarchitecture and myofiber identity were carefully examined. EOM sections from *obscna^+/^^−^;obscnb^+/^^−^* and *obscna^−^^/^^−^;obscnb^−^^/^^−^* of 10-month-old zebrafish were immunolabeled with S58 and F310 to identify all slow and all fast skeletal muscle myofibers, respectively.^46^ Additionally, phalloidin which labels actin filaments in all myofibers (Figs. 4A, 4C) and laminin (Figs. 4A–H), which labels the myofiber contours were used. Notably, the *obscna^−^^/^^−^;obscnb^−^^/^^−^* mutant zebrafish EOMs were almost devoid of slow myofibers labeled by S58 (Figs. 4B, 4D–F). As a positive control, we identified slow (S58) positive labeling in masticatory muscle myofibers in the same tissue section in the direct proximity of the EOMs (Figs. 4B, 4D, arrowheads). The myofiber proportions were quantified relative to the total number of myofibers in the EOMs. The proportion of slow myofibers (Fig. 4I, *P* < 0.0001) and fast myofibers (Fig. 4J, *P* = 0.0088) were both significantly lower in *obscna^−^^/^^−^;obscnb^−^^/^^−^* mutants compared to *obscna^+/^^−^;obscnb^+/^^−^* controls. However, the total number of myofibers was not significantly changed (Fig. 4K), suggesting a myofiber type shift rather than loss of myofibers, confirmed by TUNEL assay (Figs. 4L-N, p = 0.0143). In addition, in 95% (19/20) of the examined cross sections on *obscna^−^^/^^−^;obscnb^−^^/^^−^* double mutants, the subsarcolemmal compartment appeared enlarged compared to sibling controls (Figs. 4A, 4C, 4L, 4M).The proportion of S58 positive slow myofibers in trunk muscle of *obscna^+/^^−^;obscnb^+/^^−^* and *obscna^−^^/^^−^;obscnb^−^^/^^−^* zebrafish was similar (Supplementary Figs. S1A-B), indicating that this loss is EOM specific. To assess the impact of loss of obscurin on myofiber type identity at early age, transgenic *Tg(mylz2:*GFP*)* and *Tg(smyhc1:*tdTomato) zebrafish larvae were used to identify fast and slow myofibers, respectively.^46^^–^^48^ At 5 dpf, which corresponds to the early stage of EOM formation, both fast and slow myofibers were found in similar proportions in the EOMs of *obscna^+/^^−^;obscnb^+/^^−^* and *obscna^−^^/^^−^;obscnb^−^^/^^−^* zebrafish (Figs. 5A–E). These data indicate a myofiber type switch in both slow and fast identity. Additionally, our data suggest that this adaptation occurs gradually, as zebrafish larvae lacking obscurin appeared normal and showed no signs of lacking slow myofibers, and some S58 positive myofibers were still present in some of the examined *obscna^−^^/^^−^;obscnb^−^^/^^−^* EOMs.

### Myofibers Express the Cardiac Specific Isoform Myosin Heavy Chain *Myh7*

As some of the *obscna^−^^/^^−^;obscnb^−^^/^^−^* EOM myofibers lacked the epitopes recognized by the antibodies used to identify all conventional slow and fast myosin isoforms (S58 and F310), we hypothesized that cardiac specific MyHC genes may be expressed instead, as cardiac MyHC isoforms have previously been identified in EOMs.^49^^–^^51^ To determine whether cardiac MyHC isoforms were present in wild type zebrafish larvae, we generated antisense probes corresponding to the gene products of the zebrafish cardiac myosin genes *myh7*, *myh7bb* and *myh7*-like (*myh7l*) genes, analyzed 5 dpf wild type zebrafish larvae by whole mount *in situ* hybridization and found that *myh7* was strongly expressed in the zebrafish EOMs and in the developing heart (Figs. 5F-H). Additionally, we performed qPCR analysis in *obscna^+/^^−^;obscnb^+/^^−^* and *obscna^−^^/^^−^;obscnb^−^^/^^−^* to assess the mRNA expression of the above-mentioned genes and found that *myh7* was significantly upregulated (*P* = 0.0285) in *obscna^−^^/^^−^;obscnb^−^^/^^−^* compared to sibling controls (Figs. 5I–K). Zebrafish Myh7, also known as *vmhc*, has recently been proposed as an orthologue of human cardiac α-MyHC or MYH6.^52^ In zebrafish, Myh7 is an embryonic/juvenile cardiac specific myosin isoform expressed until 13 weeks after fertilization in zebrafish juvenile cardiac tissue.^53^ In adult zebrafish *obscna^+/^^−^/obscnb^+/^^−^* controls, *myh7* expression was identified in a small subgroup of small diameter myofibers localized at the periphery of EOMs, never overlapping with S58 labeled slow myofibers (Figs. 5L–O). In contrast, in *obscna^−^^/^^−^/obscnb^−^^/^^−^* EOMs, *myh7* expression was comparably more abundant (*P* < 0.0001) in myofibers positioned in a pattern resembling the S58 labeled myofibers in *obscna^+/^^−^;obscnb^+/^^−^* controls (Figs. 5P–T). In summary, we propose that there is a myofiber identity shift from slow and fast to *myh7* expressing myofibers in *obscna^−^^/^^−^;obscnb^−^^/^^−^* mutant EOMs.

### Lack of Obscurin Significantly Alters OKRs of Zebrafish Larvae but not of Adult Zebrafish

**Figure 6.**
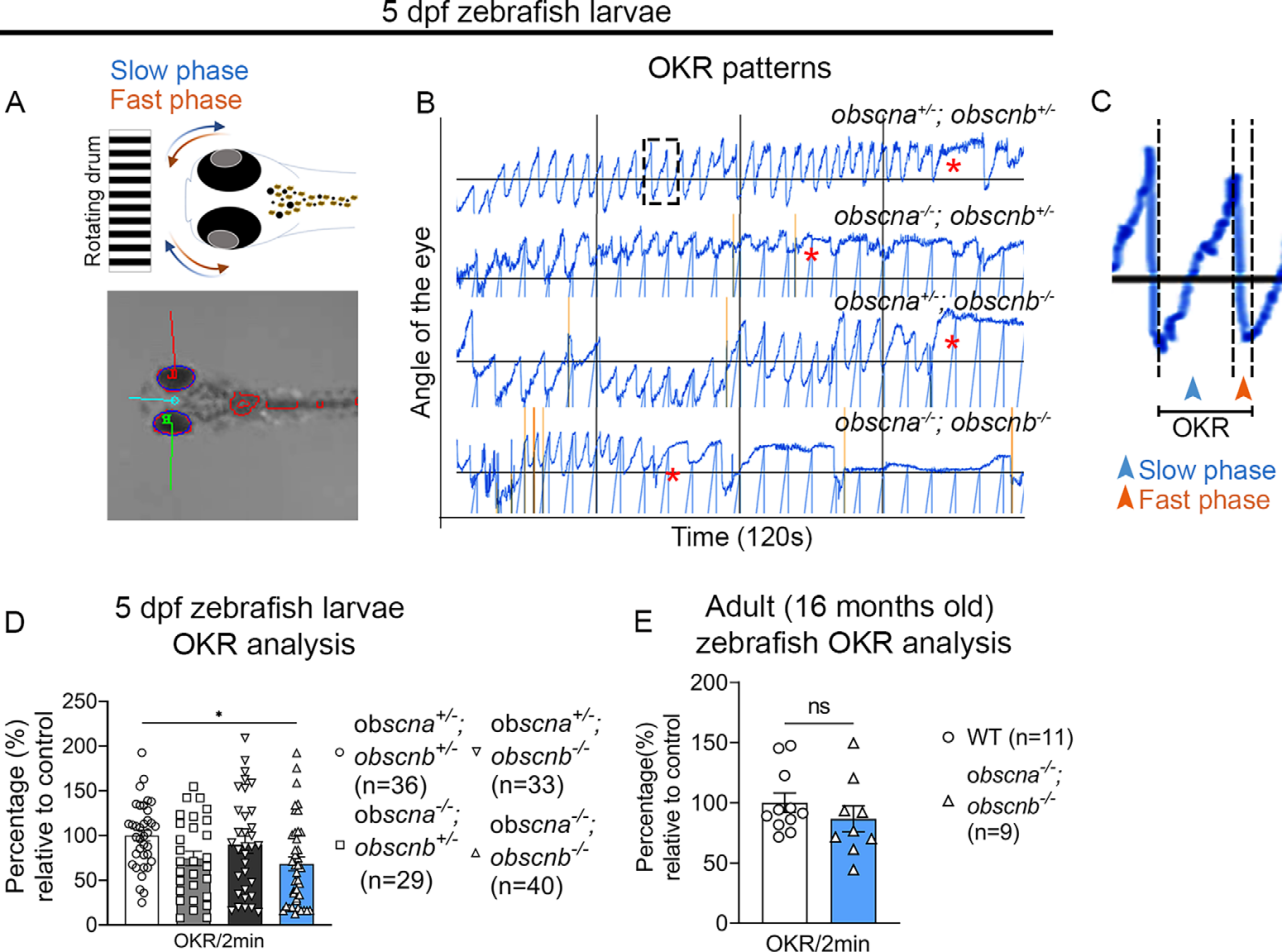
Optokinetic response analysis of the zebrafish EOMs: (**A**) Schematic representation of slow phase (blue) and fast phase (red) of the zebrafish eye. (**B**) Representative OKR patterns of *obscna^+/^^−^;obscnb^+/^^−^*, *obscna^−^^/^^−^;obscnb^+/^^−^*, *obscna^+/^^−^;obscnb^−^^/^^−^* and *obscna^−^^/^^−^;obscnb^−^^/^^−^* over 120 seconds. (**C**) A magnified image of WT OKR pattern showing slow and fast phases representing one OKR count. (**D**) Percentage of OKRs relative to control in 5 dpf zebrafish larvae. (**E**) Percentage of OKRs relative to controls in 16-month-old adult zebrafish.

To evaluate the impact of loss of obscurin in the motor function of zebrafish EOMs and trunk muscle, we performed optokinetic reflex (OKR) analysis, as well as spontaneous swimming analyses in 5 dpf zebrafish larvae and in 16 months old adult zebrafish. At 5 dpf *obscna^−^^/^^−^;obscnb^−^^/^^−^* double mutants performed significantly fewer OKRs (p = 0.018) for 120 seconds compared to *obscna^−^^/^^−^, obscnb^−^^/^^−^* single mutants and sibling heterozygous controls (Figs. 6A-D). However, we found no significant difference in the number OKRs of the adult *obscna^−^^/^^−^;obscnb^−^^/^^−^* zebrafish compared to controls (Fig. 6E). We found no significant difference between any of our *obscurin* mutant variants in spontaneous swimming tests compared to sibling controls in both age groups (Supplementary Figs. S2A-D). Additionally, 4 dpf zebrafish larvae were placed at different concentrations of methyl cellulose in 1x E3 medium (0.6%, 0.8% and 1%) and incubated at 28.5°C overnight to examine myofiber integrity after resistance swimming. The following day, 5 dpf larvae were analyzed using birefringence^54^ and no myofiber damage was observed in *obscurin* double mutants and sibling controls (Supplementary Fig. S3) indicating that obscurin has more direct impact on EOMs than trunk muscle of zebrafish.

## Discussion

In this study, muscles from human and a zebrafish in vivo model were used to evaluate a putative role for obscurin in the extraocular muscles. In both species, obscurin was located mainly at the sarcomeric M lines, which is in consonance with previous reports.^20^^,^^55^^–^^57^ By using the zebrafish in vivo model, we show that EOM function is altered in the larvae when obscurin is lacking. This indicates that even though certain specialized EOM myofibers are naturally designed to function without obscurin, the vast majority of EOM myofibers rely on obscurin to execute their roles properly. We found that the OKR patterns were less affected when only one of the two zebrafish *obscurin* genes were mutated, but when both *obscurin* genes were knocked out, the OKR pattern was severely impaired in the larvae. This clearly shows that there is partial redundancy between the two zebrafish *obscurin* genes. However, even in the absence of obscurin the myofibrils in the EOMs were apparently structurally well organized.

Here, we found that the subcellular distribution of desmin was altered and only localized subsarcolemmally in the majority of the myofibers lacking obscurin in EOMs. To the best of our knowledge, there is no proof that obscurin and desmin are directly linked in muscle tissue. Consequently, two possibilities thus arise: first, we might have found an obscurin-desmin interaction unique to EOMs. Second, the altered desmin distribution results from secondary effects of obscurin depletion. Subsarcolemmal desmin distribution was previously described to be unrelated to conventional myofiber class in EOMs of wildtype zebrafish,^9^ where this desmin pattern was found in an equal proportion of slow and fast myofibers. Here, we observed that lack of obscurin resulted in redistribution of desmin from the sarcomeric to the subsarcolemmal compartment, and this likely resulted in the enlargement of the subsarcolemmal region in cross sections of *obscurin* knockout zebrafish EOMs. This shows that there are secondary characteristics, in addition to the expression of a specific myosin heavy chain, that may be used to assign a myofiber to its identity or class. Our data strongly suggest that lack of obscurin drives the EOM myofibers toward subsarcolemmal desmin distribution and that a subset of myofibers, in particular the slow-twitch types are replaced by myofibers containing *myh7*. Hence, the changes in myofiber type and desmin characteristics observed in *obscurin* mutant EOMs are more likely due to secondary effects rather than a disturbed direct link between desmin and obscurin.

Myofiber type shift is a well-known response in trunk muscle as an adaptation mechanism to increased load, loss of ambulance or muscle atrophy.^58^ This change is however usually directed towards the type of activity involved. Interestingly, we instead found evidence for a myofiber type shift, towards an isoform mainly expressed during embryonic and juvenile stages.^53^ We did not observe myofibers with centrally positioned nuclei in EOMs lacking obscurin, which suggests that the *myh7* upregulation and loss of slow/fast positive myofibers likely is a gradual functional adaptation rather than the result of late regeneration. Furthermore, our OKR analysis showed that EOMs lacking obscurin initially struggle to perform at the same level as sibling control EOMs, however, this phenotype does not persist until adulthood. Overall, these results indicate an EOM specific adaptation to lack of obscurin during growth which compensates for the functional deficiencies at larval stages. It has previously been reported that alterations in myosin heavy chain expression in *pitx2* mutant mice where slow- MyHC myofibers of the EOMs were reduced and replaced by other MyHC expressing myofibers.^49^^,^^59^ Recently, myofiber changes in EOMs have also been observed in dystrophin null mice and human ALS patients,^60^^,^^61^ suggesting that myofiber type shift is a general coping strategy of the EOMs.

This study identified a novel role for obscurin in the EOMs. Our model will facilitate further studies in conditions related to obscurin. More data is needed particularly regarding the EOMs in patients with defects in the *obscurin* genes. In summary, we suggest that obscurin is needed to maintain myofiber identity specifically in the EOMs, and that the shift toward *myh7*-positive myofibers observed is a coping mechanism in this process.

## Supplementary Material

Supplement 1

Supplement 2
