# Supplementary material for: Obscurin Maintains Myofiber Identity in Extraocular Muscles
Source: Invest Ophthalmol Vis Sci. 2024 Feb 9;65(2):19. doi: 10.1167/iovs.65.2.19 (PMC10860686; doi:10.1167/iovs.65.2.19)
Supplement: Supplement 1 [file iovs-65-2-19_s001.pdf]

Supplementary Information

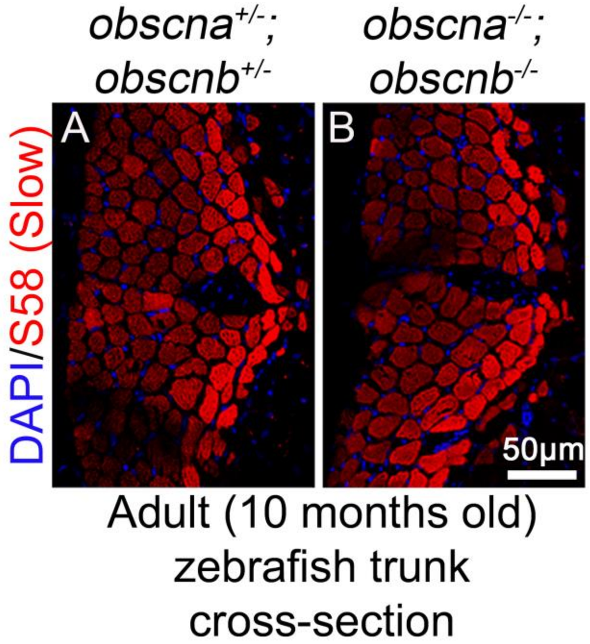

Supplementary Figure 1: **Trunk muscle myofibers analysis in *obscurin* mutant zebrafish:** (A-B) Immunolabeling of 10 months old adult zebrafish's cross-sections of trunk muscle using slow myofiber marker, S58 (in red) exhibiting no difference between the *obscurin* double knockout and the sibling control.

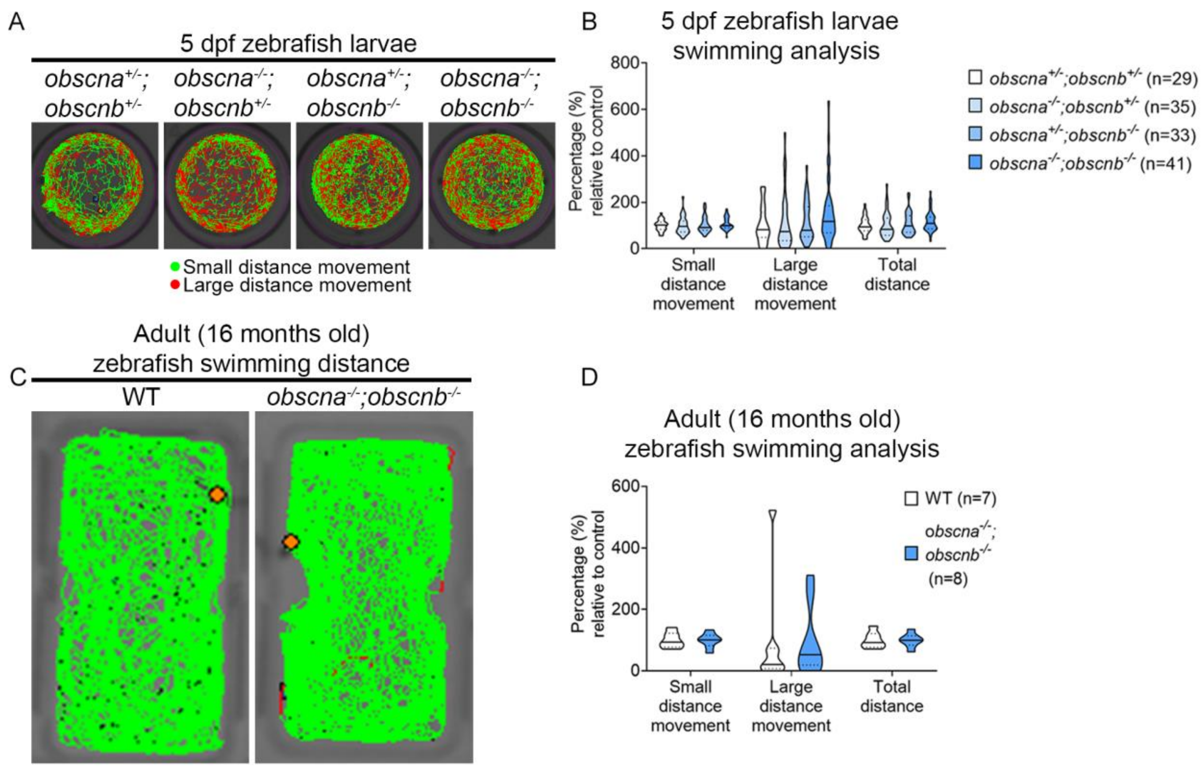

Supplementary Figure 2. Swimming analysis: (A-D) Swimming distance to examine trunk muscle function in the absence of Obscurin in larvae and adult zebrafish. Representative images of spontaneous swimming tracks for 1h of (A) *obscna*$^{+/-}$*; obscnb*$^{+/-}$, *obscna*$^{-/-}$*; obscnb*$^{+/-}$, *obscna*$^{+/-}$*; obscnb*$^{-/-}$, and *obscna*$^{-/-}$*; obscnb*$^{-/-}$ in 5 dpf zebrafish larvae, and (C) wild type and *obscna*$^{-/-}$*; obscnb*$^{-/-}$ in 16 months old adult zebrafish (green, low velocity; red, high velocity). (B, D) The relative percentage of small distance, large distance and total distance swam by the different genotypes in (B) 5 dpf zebrafish larvae and (D) 16 months old adult zebrafish. No significant differences were found between the WT and the *obscna*$^{-/-}$*; obscnb*$^{-/-}$ zebrafish.

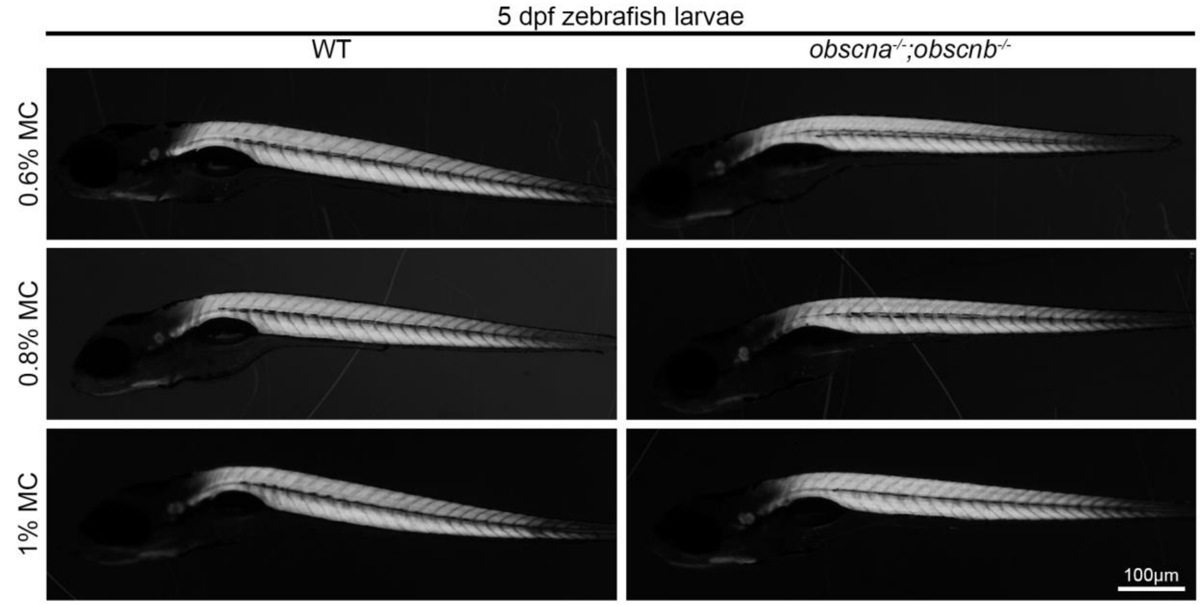

Supplementary Figure 3. Analysis of birefringence in methylcellulose-treated larvae. Wild-type and *obscna*$^{-/-}$; *obscnb*$^{-/-}$ zebrafish larvae were incubated at different concentrations of methylcellulose (0.6%, 0.8% and 1%, n=10 in each group) from 4 to 5 dpf. Representative birefringence images of wild type (left) and *obscna*$^{-/-}$;*obscnb*$^{-/-}$ double mutants (right) at 5 dpf are shown.

Supplementary Video 1. Adult zebrafish OKR. Representative video showing OKR of 16 months old adult wild type zebrafish.

Supplementary Table 1. Primary antibodies

**Primary antibodies**	**Host species**	**Dilution**	**Source**	**Antibody registry ID**
Obscurin IQ	Rabbit	1:100	Gift from Prof. M. Gautel	NA
Acetylcholine receptor gamma (AChRγ)	Mouse	1:5	Gentex	GTX74890

F310	Mouse	1:10	DSHB	AB_531863
S58	Mouse	1:10	DSHB	AB_528377
Desmin	Rabbit	1:100	Abcam	ab15200
Laminin	Rabbit	1:100	Sigma	L9393
Laminin	Sheep	1:6000	Binding site group, Bermingham, UK	PC128
Myomesin(B4)	Mouse	1:5	DSHB	AB_760349
Tenascin (M1-B4)	Mouse	1:100	DSHB	AB_528488

*DSHB, development studies hybridoma bank

Supplementary Table 2. Secondary antibodies

**Secondary antibody**	**Dilution**	**Source**	**Antibody registry ID**
Alexa Fluor 488 donkey anti-mouse IgG	1:300	Jackson ImmunoResearch	AB_2313584
Alexa Fluor 488 donkey anti-rabbit IgG	1:300	Jackson ImmunoResearch	*AB-2313584*
Rhodamine Red™-X donkey anti-rabbit	1:500	Jackson ImmunoResearch	AB_2340613
Rhodamine Red™-X donkey anti-mouse	1:500	Jackson ImmunoResearch	AB_2340832
Rhodamine Red™-X donkey anti-sheep	1:500	Jackson ImmunoResearch	AB_2340737
Alexa Fluor 647 goat anti-mouse IgG	1:300	Invitrogen, Molecular probes	A32795
Alexa Fluor 647 goat anti-rabbit IgG	1:300	Invitrogen, Molecular probes	A32787

Supplementary Table 3. Directly conjugated high affinity probes

Alexa Fluor 488 Phalloidin directly conjugated	1:100	Invitrogen, Molecular probes	A12379
Rhodamine Phalloidin directly conjugated	1:100	Invitrogen, Molecular probes	R415
Alexa Fluor 647 Phalloidin directly conjugated	1:100	Invitrogen, Molecular probes	A22287
α- Bungarotoxin Alexa Fluor 647 directly conjugated	1:100	Invitrogen, Molecular probes	B35450
